# Case of Rupture of the Uterus, in Which the Operation of Gastrotomy Was Successfully Performed by John Neill, M. D., Surgeon to the Pennsylvania Hospital

**Published:** 1854-10

**Authors:** John K. Mason

**Affiliations:** Philadelphia


### MEDICAL EXAMINER.

NEW SERIES.—NO. CXVIII.—OCTOBER, 1854.

---

### ORIGINAL COMMUNICATIONS.

---

*Case of Rupture of the Uterus, in which the operation of Gastrotomy was successfully performed by John Neill, M. D., Surgeon to the Pennsylvania Hospital.* By JOHN K. MASON, M. D., of Philadelphia.

Monday, 24th of July, called to Mrs. John McDevitt, South above 20th street, in labor with her sixth child; reached her at 9, P. M.; had been in active labor for about an hour; of a florid complexion, somewhat fleshy, large muscular development, with all the appearance of possessing a constitution of more than ordinary strength and vigor.

Upon examination, found the os uteri about half open, membranes presenting unbroken, the head to be felt high up above the superior strait; the pains were good, but by no means violent, with distinct remissions of five or six minutes; left her, and returning in about an hour, found the os uteri fully open, pains somewhat stronger, but still the presenting part did not descend. I now ruptured the membranes in the expectation that, as there was neither contraction nor rigidity, the head would come down into the pelvis without further delay. In this I was disappointed, and it was evident that the head had some difficulty in entering the superior strait. Still it advanced a little, and I thought I could detect the anterior fontanel looking towards the left acetabulum, giving the fourth position of Baudeloque. I was, however, by no means certain on this point, but resolved to wait. I made a visit in the next street, and returned to the patient in less than half an hour; the pains were now much stronger, and I thought that the head had advanced slightly. At this time Mrs. M. was obliged to get up, for the purpose of relieving her bowels, and I went down stairs, still without the slightest anxiety as to the result of the labor, for the spirits were good, the countenance cheerful, the woman well formed and vigorous, and a state of active labor had not existed for more than two hours and a half.

While at stool, the patient had two pains; during the latter she suddenly complained of intense agony, with a burning sensation in the right side; the woman hurried her to bed, and called me into the room; I found her on her back in great torture, which she assured me was no longer the pain of labor, but that something had gone wrong inside of her.

I examined her pulse and found it but little altered; this, added to the circumstance of their being neither vomiting nor cold clammy skin, nor any thing like an approach to syncope made me hope that matters were not so bad as I had at first apprehended; but after administering some forty drops of laudanum, using hot fomentations, and waiting for some time, finding that the uterine contractions were completely suspended, that the presenting part had receded, and that there was a sanguineous discharge, though not profuse from the vagina, I felt convinced that the uterus was ruptured.

Before, however, proposing any operation, I called upon Dr. Hollingsworth for his advice and assistance. He immediately came in the kindest manner, and after careful investigation, the diagnosis was distinctly made out. The placenta had *not* passed into the cavity of the abdomen, for it could be distinctly felt with the cord passing from it. The head of the child could be detected through the abdominal parietes occupying the lower part of the abdomen on the right side, near the inguinal region, but no portion of it remained in the uterus.

Under these desperate circumstances, we deliberated with sad forebodings on the treatment to be adopted, in order, if possible, not to lessen the little chance of life remaining. I am aware that all the best authorities recommend introducing the hand through the torn womb, into the bowels of the victim, seizing the feet, and first dragging the infant back into the womb, and from thence, per vias naturales, into the world. They say that it gives the child a better chance ; it may be so, I am not prepared to dispute that point, for I thank God I have had no experience in such a procedure ; but this I do know, that the description of the operation has always filled me with unutterable horror.

Another thing, which I am bound to confess, though it may appear very unprofessional and unnatural to some, is, that I never thought of the child or its life, or anything about it, except to wish, that as it had pleased God to place it there, it would please him, in his infinite mercy, to assist me in getting it away, without tearing the poor devoted woman to pieces, and entailing upon myself the terrible conviction, that I had made almost certain death a certainty.

After due consideration, however, we determined to explain the nature of the necessary operation, by turning, to the patient, and propose it as a dernier resort. This we did, but she absolutely refused to submit to it ; and from the hydrocephalic condition of the head, afterwards ascertained, we had reason to be thankful that she did so. At this time, there was no vomiting, the expression of her countenance was good, the pulse firm, and the skin natural ; the pain in the abdomen, at first very severe, had now much abated ; and after administering a powerful dose of morphia we left her, determining to see her in the morning, and then be guided by circumstances.

Next morning, the 25th, when Dr. Hollingsworth and myself visited her, we found her much better than we anticipated ; pulse firm and strong, countenance bright and mind unclouded ; longer to leave her undelivered was out of the question ; professional duty and common humanity, alike demanded that an effort, however desperate, should be made to save her. We, therefore, determined to propose gastrotomy, as that operation, in our opinion, afforded her the best chance ; to this, encouraged by feeling better than she anticipated, she at last consented, and after consulting Dr. Neill, who undertook the performance of the operation, it was determined on.

*Operation by Dr. Neill.*—The patient was placed upon a stout table covered with blankets, her shoulders and head supported by pillows; and as a preliminary step, about four ounces of ether were administered by inhalation. The incision was made in the linea alba, commencing about two inches below the umbilicus, and extending towards the pubis full six inches; the moment the opening was made, large quantities of mingled blood and clots escaped, the omentum seeming saturated with blood, and both the visceral and parietal peritoneum being deeply stained. A dead child's back presented, its head lying low down towards the right groin, its feet to the left; it was immediately removed and found to be hydrocephalic, the bi-parietal diameter of the head measuring, I should suppose, six inches, the occipito frontal probably seven. Its entire weight I should judge to be not less than ten pounds.

The rent in the uterus appeared to be enormous, and perfectly uncontracted, for the operator passed both hands through it, right down into the organ, and, as it were, scooped up the placenta with all the coagula within his reach. Upon the removal of his hands, the womb instantly contracted to about the size of a man's fist. The blood, fluid as well as coagulated, was then removed from the cavity of the abdomen as far as practicable, disturbing the viscera as little as possible. The incision was then closed by five sutures, and afterwards by long adhesive straps, leaving an opening at the lower part of the wound to favor the escape of fluids; a compress and binder completed the arrangement.

The patient's strength was less exhausted than could have been anticipated; spirits good; pulse 120—firm and equal; the time occupied by the whole operation did not, I should think, exceed five minutes. In half an hour she was placed in bed, and an enema of laudanum administered; grain doses of opium were directed to be given by the mouth every three hours, in order to keep her, if possible, in a perfect state of repose, and prevent any action of the abdominal viscera; at the same time the system was supported by nourishing fluids, beef tea, &c.

Shortly after the operation the patient began to vomit a greenish watery fluid, which continued several hours, but was checked by the exhibition of small quantities of brandy with ice; the opium treatment seemed to agree with her, for she slept and complained of but little pain.

On the morning of the 26th I visited her in company with

Dr. Hollingsworth. Found her tolerably easy; mind cheerful; tongue clean and moist; pulse 120; the abdomen was tympanitic and very much distended; the breathing much embarrassed by the accumulation of gas. Ordered her to continue the opium pills and to have an injection containing turpentine.

On making my evening visit I learned that the bowels had been slightly opened, and that she had passed large quantities of flatus, by which the tympanitic distension of the abdomen was much lessened; breathing easy and natural; pulse rapid and weak. Directed brandy to be continued with the opium.

27th and 28th.—Continued in much the same condition—occasionally vomiting.

On the 29th Dr. Neill visited her with Dr. Hollingsworth and myself. Removed the stitches from the wound, which was healthy and closing extremely well; she was now ordered milk punch, ad libitum.

At this time there was a very copious, dark, offensive discharge from the vagina, which was kept continually syringed with warm water and soap. The bowels had been moved once copiously; pulse 120; tongue moist, but slightly furred. The patient looked so hopeful and strong, that we began to feel en couraged.

On the morning of the 30th I understood that she had passed a restless night. Looked very much worse; the lips were pale; countenance dejected; pulse 130; vomiting of green matter without effort, in fact a regurgitation of the fluids contained in the stomach.

I began to lose hope. Still her mind never wavered—day nor night—and when spoken to she replied quickly and clearly, but without anything like an unnatural elevation, a condition which I have sometimes observed in bad cases of uterine phlebitis. The discharge from the vagina was less copious and less offensive.

When I saw her in the evening she was laboring under the worst possible symptoms; so much so, indeed, that I thought it possible she might die before the morning. Her pulse was from 135 to 140—very weak; her feet and legs were cold, also the lower part of the belly; her wrists and arms to the shoulders in the same condition, and bedewed with a clammy sweat.

I confess I regarded her as moribund, and the priest in attendance told her that she was dying and must make her peace with God; the poor woman replied that she would make her peace with God most willingly, but that the Revd. Father was wrong, that she was not dying yet, she did not feel like dying. And as it proved, she was right, for the next morning, the 31st, I was agreeably surprised to find that her skin had regained its natural temperature, that her strength had improved, and that she was altogether better than on the previous day. There was no pain on pressure of the abdomen, but still there was considerable tympanites, and the pulse continued at 135.

On the first of August the vomiting continued, but only occasionally.

On the 2d, vomiting had ceased entirely. I watched with great anxiety for a diminution in the frequency of the pulse, as indicating some favorable change, but as yet in vain.

Notwithstanding the steady pursuance of the opiate treatment, the patient's bowels, on the 1st, were largely opened, three or four times. She complained of great pain, before each evacuation; opiate enemeta checked this, and on the 2nd she had but one stool, perfectly natural in color, and of the consistence ordinarily produced by a dose of castor oil.

August 3d.—Pulse somewhat slower—about 120; dressed the wound in the abdomen; did not think it looked quite so well; some discharge from one of the suture openings; was suffering from great uneasiness of the bowels, they having been opened several times; before each movement considerable pain was complained of, somewhat resembling the tormina of dysentery—color perfectly natural. In the evening there again appeared great coldness of the extremities. Ordered an enema of starch and laudanum, with hot bricks to the legs and feet, brandy and milk to be given freely.

On the fourth, found the patient warm, pulse 130, tongue foul, complaining of great pain in the bowels, which had been moved several times during the night, the breathing high and labored. Both Dr. Neill and myself thought her prospect of recovery worse than usual. Dr. Hollingsworth had left town, and I was obliged to be absent from the city for some hours, Dr. Neill therefore undertook to see her for me. In the evening, I found her symptoms the same as in the morning; ordered her a large teaspoonful of laudanum, as an injection; and desired the attendant to give her all the nourishment she could take, with a continuance of the brandy and milk.

Saw her, with Dr. Neill, on the morning of the 5th, breathing decidedly improved, countenance and spirits better, though the pulse was weak and continued at 130; tongue cleaning; dressed the abdominal wound, which looked much healthier, though there was considerable discharge from another of the suture wounds; had passed a comfortable night, slept well, and had had no pain nor trouble with her bowels; but there had been a considerable discharge from the womb, described by the nurse as being of a clear red color, and devoid of smell.

At half past nine in the evening saw her again, condition unchanged, pulse the same, uterine discharge still copious, bowels had been opened once.

On the 6th, found her much improved, pulse 120, tongue clean, expression of face natural, heat of skin almost natural, with very little thirst, the abdominal wound nearly healed. In the evening, found her easy, but showing more weakness. This I attributed to the uterine discharge, which continued copious.

Morning of the 7th, stronger and better, pulse 110, discharge from the womb much lessened; had slept soundly all night. At ten o'clock, evening of the same day, great change had taken place; pulse 100, firm and steady, uterine discharge nearly suppressed; had taken her food regularly and with appetite; bowels had been opened once naturally during the day.

From this time she improved so rapidly, that on the 15th, she came down stairs; on the 24th, just a month from the time of the rupture, was at the wash-tub; and on the 2nd of September, I met her in the street, when she told me she felt as well as she did before the accident.

The treatment of this fatal and distressing accident has been the subject of much difference of opinion among the learned in the obstetrical art; some advocating immediate delivery by version or by the forceps, when practicable, or by gastrotomy; while others have expressed themselves in favor of leaving the patient entirely to the recuperative powers of nature; contenting themselves by recommending their pupils to make no effort at delivery, but merely to combat symptoms as they arose; and any one who takes the trouble to read Dr. Trask's valuable monograph on the subject, with his series of cases, (*See Amer. Jour. of Med. Science, January and April,* 1848.) will find that each mode of practice has occasionally succeeded.

But the object to be gained by a numerous array of cases with their results is a knowledge of the mode of management the least likely to increase the danger, and, therefore, the most likely to eventuate in recovery. It is now pretty generally conceded, that the chances of this fortunate result are materially promoted by speedy delivery; either by turning, by the forceps, or by gastrotomy, according to the peculiar circumstances attending each particular case.

To the zeal and industry of Dr. Trask, of Brooklyn, we are indebted for the accumulation and results of 303 cases, comprising rupture from mechanical violence; rupture occurring during gestation, and rupture at the full term; rupture likewise in every degree of severity, partial or complete; in some instances, not involving the peritoneum, and in some cases, confined merely to the neck of the womb.

In this series, there are 128 recoveries.

The following table shows an evident preponderance of fortunate results in favor of gastrotomy.

| | | |
|---|---|---|
| Gastrotomy, - - - - | saved, 18 - - | lost, 7. |
| Undelivered, - - - | saved, 18 - - | lost, 50. |
| Other modes of delivery, | saved, 28 - - | lost, 51. |

Dr. Trask makes the following remarks on these results: "We wish to be distinctly understood, that we do not consider that our statistics afford us the actual proportion of recoveries and deaths after the respective modes of treatment.

Few are ambitious to publish unsuccessful cases, and hence the reason of so large a proportion of successful terminations as our series exhibits. But we see no reason why such apparent success should follow gastrotomy, were not its results in reality, in the main, more satisfactory than that of the other modes of delivery. A degree of eclat attaches to its performance, even if it be unsuccessful, far more than to the application of the forceps, to perforation or to version; therefore, there can be no causes tending to suppress the publication of unsuccessful cases of gastrotomy, which do not apply in an equal and ever greater degree to the other modes specified." With equal truth it may be observed, that the proportion of recoveries in 303 cases, viz., 128, must not, in this fatal accident, be taken even as an approximation to the true proportion of deaths and survivals. We, ourselves, know of three cases of rupture of the womb occurring in this city, which have never been reported, and by making inquiry among the members of the profession, we have no doubt but that many more could be found. The reasons for this silence are obvious. Many are so much engaged that they cannot, or will not, give the time for drawing up a careful report; others, particularly those practising among the lower orders of the community, are afraid of the blame which might, on such an occasion, attach, however unfairly, to themselves, and cases, no doubt, have occurred where the accident has not been detected during the life of the patient, and where a post mortem has not been sought by the physician, or, if requested, has not been granted, and the case has been certified simply as one of sudden death during labor, from exhaustion, disease of the heart, pulmonary coagula, shock, syncope, &c., &c.

But these statistics, though useless as a means of estimating comparative results, are, by the accumulation of so many cases of recovery, highly useful and important. They prove the perfect fallacy of the old doctrine, maintained by such high authority as that of Hunter, Burns and Denman, that the most humane course was to leave the patients undelivered, and trust entirely to the resisting and recuperative powers of nature. They prove incontestibly, what Dr. Dewees has so earnestly asserted, viz., that the do-nothing advocation is opposed to the best interests of humanity; for such cases so treated have almost invariably perished, while they demonstrate, beyond the possibility of cavil, that immediate delivery by one means or another, has frequenlty succeeded under the most desperate and uncompromising circumstances.

On the subject of delivery, or non-interference, Dr. Trask gives us the subjoined results:

"Of 154 cases delivered by artificial means, 97 *died;* 57 *survived.*

Of 89 abandoned undelivered, 65 *died* and 24 *survived.*

Of 31 delivered by natural efforts, 20 *died* and 11 *survived,*

and these include, in both instances, cases of rupture of the os, in which the peritoneum was not involved.

Of 6 in whom artificial delivery was tried and failed, all died undelivered.

A comparison of those delivered by art and of those abandoned undelivered, yields 37 of the former, as saved, to 27 of the latter in the hundred, showing that the chances in the former case are considerably better than in the latter."

In looking over some of the periodicals, we have collected the following additional cases of ruptured uterus, published ulterior to the date of Dr. Trask's memoir:

Cases of rupture of the uterus have recently been reported by Mr. Brownhill, *Prov. Journal, Dec. 29th*, 1849, and by Dr. Smallwood, U. S., *Brit. Amer. Journ., Jan.*, 1848. Both were fatal. Mode of treatment not mentioned.

Two cases of rupture of the uterus are reported by Dr. Mitchell. In the first, one leg had escaped into the abdomen; the child was extracted, and the woman recovered under large doses of opium.

In the second case, the escape of the child was complete; gastrotomy was performed after the mother's death, but it is not stated whether the child was saved, though it may be inferred that it was not.—*Obstetrical Record, No.* 13.

A case of recovery after ruptured uterus is also recorded by Dr. Coley. In this instance the fœtus, with the exception of the head, had escaped into the peritoneal cavity. The narrator states that he introduced his hand through the rent, and seizing the feet dragged the body back into the uterus, and so delivered the child.—*Ibid, No.* 11.

Dr. Simpson also describes two cases of rupture depending on hydrocephalic fœtuses, and alludes to the frequent connexion between the two conditions. In confirmation of this he states, that out of 74 cases of intra uterine hydrocephalus collected by Dr. Keith, rupture of the uterus occurred in 16. He further insists on the necessity of immediate delivery when the fœtus is ascertained to be hydrocephalic, and advises puncture of the cranium with a trocar rather than craniotomy.—*Monthly Journ., June*, 1848.

A case of recovery from rupture of the womb, is reported by

Mr. Church, in the Lancet, May 29th. The child was extracted dead, the mother made a good recovery.—*Ranking's Abstract, Jan.* 1849.

A case of extreme rupture is reported by Mr. Long. The child was completely thrown into the cavity of the abdomen. Gastrotomy was performed after some little delay. The operation was borne well, but the woman sank, and died on the third day. No post mortem permitted. Notwithstanding the magnitude of the injury here sustained, Mr. Long was convinced that had the patient enjoyed the benefits of a regulated temperature and the other advantages of regular attendance which a hospital might have supplied, she would have survived.—*Dublin Medical Press, April* 17*th*, 1850.

Mr. Stobo has recorded an instance of recovery after rupture of the uterus. The nature of the accident was indisputable, as the author felt the intestines through the rent. No mention is made of the child; we, therefore, conclude, that it did not survive.—*Medical Times, April* 6*th*, 1850.

A remarkable, and, perhaps, unique example of rupture of the uterus, is narrated by Mr. Stolty, in which the entire ovum escaped into the abdomen at full term. The woman died of hemorrhage.—*Gazette Medicale de Strasbourg.*

[Note.—We believe that the above is the only instance in the United States, in which gastrotomy has been successfully performed, in case of rupture of the uterus. In Dr. R. Estep's case, reported by Wm. Bowen, M. D., in the Amer. Jour., Oct. 1843, gastrotomy, was performed for the delivery of a severed head, the body of the child having been previously extracted, per vias naturales. As, however, there existed but a slight rent in the uterus, which had to be enlarged to the extent of five or six inches, the case should be termed, we think, one of gastro-hysterotomy or Cæsarean section.—Ed. Med. Ex]

